# High GPER expression in triple-negative breast cancer is linked to pro-metastatic pathways and predicts poor patient outcomes

**DOI:** 10.1038/s41523-022-00472-4

**Published:** 2022-08-30

**Authors:** Ting Xu, Ding Ma, Sheng Chen, Rui Tang, Jianling Yang, Chunhui Meng, Yang Feng, Li Liu, Jiangfen Wang, Haojun Luo, Keda Yu

**Affiliations:** 1 Department of Endocrine and Breast Surgery, The First Affiliated Hospital of Chongqing Medical University, 1 Youyi Road, Chongqing, 400010 People’s Republic of China; 2 Department of Breast Surgery, Precision Cancer Medicine Center, Fudan University Shanghai Cancer Center, 270 Dong’an Road, Shanghai, 200032 People’s Republic of China; 3 Key Laboratory of Laboratory Medical Diagnostics, Chinese Ministry of Education, Chongqing Medical University, Chongqing, 400016 People’s Republic of China; 4 Department of Thyroid and Breast Surgery, Weihai Municipal Hospital, 70 Heping Road, Huancui District, Weihai, Shandong 264200 People’s Republic of China; 5 Department of Thyroid and Breast Surgery, Heze Municipal Hospital, 2888 Caozhou West Road, Heze, Shandong 274031 People’s Republic of China; 6 Department of Thyroid and Breast Surgery, The Second Affiliated Hospital of Chongqing Medical University, 74 Linjiang Road, Chongqing, 400010 People’s Republic of China; 7 Department of General Surgery, Shanxi Provincial People’s Hospital, Taiyuan, Shanxi 030000 People’s Republic of China

**Keywords:** Metastasis, Cancer genomics, Breast cancer

## Abstract

Triple-negative breast cancer (TNBC) is a particularly aggressive and heterogeneous disease with few effective targeted therapies and precision therapeutic options over a long period. It is generally considered that TNBC is an estrogen-independent breast cancer, while a new estrogen receptor, namely G protein-coupled estrogen receptor (GPER), is demonstrated to mediate estrogenic actions in TNBC. Based on our transcriptomic analysis, expression of GPER was correlated with clinicopathological variables and survival of 360 TNBC patients. GPER expression at mRNA level was significantly correlated with immunohistochemistry scoring in 12 randomly chosen samples. According to the cutoff value, 26.4% (95/360) of patients showed high GPER expression and significant correlation with the mRNA subtype of TNBC (*P* = 0.001), total metastatic events (*P* = 0.019) and liver metastasis (*P* = 0.011). In quantitative comparison, GPER abundance is correlated with the high-risk subtype of TNBC. At a median follow-up interval of 67.1 months, a significant trend towards reduced distant metastasis-free survival (DMFS) (*P* = 0.014) was found by Kaplan–Meier analysis in patients with high GPER expression. Furthermore, univariate analysis confirmed that GPER was a significant prognostic factor for DMFS in TNBC patients. Besides, high GPER expression was significantly linked to the worse survival in patients with lymph node metastasis, TNM stage III as well as nuclear grade G3 tumors. Transcriptome-based bioinformatics analysis revealed that GPER was linked to pro-metastatic pathways in our cohort. These results may supply new insights into GPER-mediated estrogen carcinogenesis in TNBC, thus providing a potential strategy for endocrine therapy of TNBC.

## Introduction

Triple-negative breast cancer (TNBC) accounts for 10–15% of all breast cancers and is characterized by the lack of expression of the estrogen receptor α (ER-α), the progesterone receptor (PR), and the human epidermal growth factor receptor 2 (HER2)^1,2^. When compared with other subtypes of breast cancer, TNBC exhibits the most aggressive course and the highest rate of early distant recurrence, especially in the lungs and brains, both of which predict death in the short term^3,4^. Notably, TNBCs occur more frequently in younger patients with its percentage increasing to 25–30% in patients under 50^1,2^. Concerning systemic therapy, chemotherapy remains the standard management for TNBC while targeting therapy is still at its early stage. Theoretically, the most effective strategy to improve patient outcomes may be by blocking the metastatic process.

Estrogen, predominantly 17β-estradiol (E2), is a critical driver of mammary development and an essential etiological factor for breast cancer. As biologic mediators of estrogenic effects, ERs are widely distributed in breast cancer. Specifically, ER-α, which is detected in about 70% of breast cancers, is used as not only a powerful prognostic factor but also an efficient target for patients^5^. The endocrine therapy that blocks estrogen signaling, either by suppressing ER-α activity or by inhibiting estrogen production, is central to the multidisciplinary management of patients with breast cancer, based not only on the significant effectiveness but also on the convenience and safety of these agents^6,7^. However, estrogen carcinogenesis, as well as endocrine therapy, were long neglected in TNBC, logically due to the absence of ER-α. It has been recently shown that alternative ERs including G protein-coupled estrogen receptor (GPER), ER-β, and ER-α36 (a variant of ER-α) can trigger estrogen-responsivity in TNBC^8,9^, which leads to a growing concern.

The identification of GPER, also known as GPR30, which was recognized as a membrane-associated receptor binding E2 with high affinity to mediate rapid and nongenomic estrogenic effects, including transactivation of epidermal growth factor receptor and production of second messengers such as cAMP, calcium and inositol triphosphate, has challenged the traditional concept stating that TNBC was estrogen-independent^10–13^. GPER is a member of G protein-coupled receptors (GPCRs), whose biological activity is dictated by posttranscriptional modifications (such as phosphorylation and ubiquitination) that control receptor concentrations at the plasma membrane^14^. In our earlier reports and others, GPER was detected in more than 60% of primary TNBC samples and several TNBC cell lines^15–20^. Importantly, GPER was linked to the metastatic behaviors of TNBC cells in vitro and in vivo. Ligands including E2 and bisphenol A were claimed to trigger GPER to promote migration and metastasis of TNBC cells^21–23^, although the designation of GPER as a cognate ER is still debated sometimes^24–27^. In a recent bioinformatics analysis, GPER was also correlated with pro-metastatic genes and pathways in ER-α negative breast cancer^28^. Considering the advantages of endocrine therapy, GPER has been included as a candidate biomarker and a potential therapeutic target for TNBC^29^. However, a controversial report concludes that GPER activation could inhibit the in vivo invasive potential of TNBC via suppression of epithelial-mesenchymal transformation^15^. Thus, the role of GPER in TNBC metastasis needs further confirmation.

As an alternative ER, GPER has caught increasing attention in breast cancer research, and the relationship between GPER and breast cancer outcomes has been addressed in multiple studies^30–34^. Controversial findings on the prognostic role of GPER as well as the association between GPER expression and clinicopathological determinants of breast cancer have been reported. For instance, GPER was linked to worse relapse-free survival (RFS) in breast cancer patients treated with tamoxifen^31^. Meanwhile, GPER was also correlated with an increased distant disease-free survival (DDFS) of ER-α positive breast cancer^32^. The biological functions of GPER largely depend on the cellular background in vitro^12,13^. In patients with TNBC, an early report provided a clue that GPER might be associated with a poorer prognosis with a trend towards increased recurrences (without statistical significance, *n* = 18)^35^. Recently, high expression of GPER was related to decreased outcomes in TNBC, including the local RFS, DDFS, overall survival (OS), and progression-free survival (PFS) in a Chinese cohort (*n* = 249)^17^. A bioinformatics analysis also associated the high expression of GPER with the decreased disease-free interval in ER-α negative breast cancer, although the scale of the cohort was limited (*n* = 120)^28^. Thus, the prognostic significance of GPER in TNBC needs to be evaluated in larger cohorts.

We have reported the largest single-center study concerning the multi-omics profiling of TNBC, delineating the genomic and transcriptomic landscape of Chinese TNBC patients^36^. In this cohort, 360 cases had RNA sequencing data on primary tumor tissue. To further evaluate the prognostic role of GPER, especially on metastatic manifestations in these patients, we analyzed the correlation between GPER expression and clinicopathological determinants of TNBC progression and long-term survival herein. We also show a bioinformatics analysis based on the transcriptomic profiles of our cohort to better understand the estrogenic carcinogenesis mediated by GPER in this aggressive breast cancer subtype.

## Results

### Patient characteristics

360 patients who underwent surgery at Fudan University Shanghai Cancer Center (FUSCC) between 2007 and 2014 were included in this study (Table 1). The mean age of all patients was 53.3 ± 11.4 years old (range, 25–84 years old) and 62.2% of them were post-menopause. Most tumors were pT2 (60.8%), without lymph node metastasis (LNM) (58.1%), TNM stage II (61.1%), invasive ductal carcinoma (91.7%), nuclear grade 3 (64.4%), and within the basal-like intrinsic subtype (76.9%). As for this cohort, we classified the tumors into four transcriptome-based subtypes: luminal androgen receptor (LAR) subtype (22.5%), immunomodulatory (IM) subtype (24.2%), basal-like immune-suppressed (BLIS) subtype (38.6%) and mesenchymal-like (MES) subtype (14.7%). Distant metastases were excluded present at the time of surgery. No patients received any systemic adjuvant therapy besides chemotherapy. The median follow-up interval was 67.1 months (range 0.3–144.2 months). At the time of analysis, 60 patients underwent recurrence, 50 patients had metastatic events and 40 patients died; the RFS was 83.3%, DMFS was 86.1% and OS was 88.9%.

**Table 1:** Tumor characteristics and GPER distribution.

Variables	No. (%)/Mean ± SD			
	Total (*N* = 360)	GPER-Low (*N* = 265)	GPER-High (*N* = 95)	*P*
Age	53.3 ± 11.4	53.4 ± 11.3	52.8 ± 11.5	0.624
Menopause status				0.832
Pre-menopause	132 (36.7)	98 (74.2)	34 (25.8)	
Post-menopause	224 (62.2)	164 (73.2)	60 (26.8)	
Tumor size (pT)	2.64 ± 1.17	2.64 ± 1.25	2.66 ± 0.92	0.891
pT1	131 (36.4)	100 (76.3)	31 (23.7)	
pT2	219 (60.8)	156 (71.2)	63 (28.8)	
pT3	9 (2.5)	8 (88.9)	1 (11.1)	
LNM (pN)				0.426
pN0	209 (58.1)	158 (75.6)	51 (24.4)	
pN1	97 (26.9)	70 (72.2)	27 (27.8)	
pN2	32 (8.9)	22 (68.8)	10 (31.2)	
pN3	17 (4.7)	10 (58.8)	7 (41.2)	
TNM stage				0.513
I	89 (24.7)	70 (78.7)	19 (21.3)	
IIA	144 (40.0)	105 (72.9)	39 (27.1)	
IIB	76 (21.1)	56 (73.7)	20 (26.3)	
IIIA	34 (9.4)	24 (70.6)	10 (29.4)	
IIIC	17 (4.7)	10 (58.8)	7 (41.2)	
Histological				0.182
IDC	330 (91.7)	246 (74.5)	84 (25.5)	
Others	30 (8.3)	19 (63.3)	11 (36.7)	
Nuclear grade				0.304
2	93 (25.8)	65 (69.9)	28 (30.1)	
3	232 (64.4)	175 (75.4)	57 (24.6)	
unknown	35 (9.7)			
Necrosis				0.027*
No	151 (41.9)	122 (80.8)	29 (19.2)	
Yes	113 (31.4)	78 (69.0)	35 (31.0)	
Ki67	52.8 ± 25.3	51.7 ± 25.2	55.8 ± 25.5	0.180
Intrinsic subtype				0.755
Basal like	277 (76.9)	205 (74.0)	72 (26.0)	
Others	83 (23.1)	60 (72.3)	23 (27.7)	
mRNA subtype				0.001*
IM	87 (24.2)	77 (88.5)	10 (11.5)	
LAR	81 (22.5)	59 (72.8)	22 (27.2)	
BLIS	139 (38.6)	97 (69.8)	42 (30.2)	
MES	53 (14.7)	32 (60.4)	21 (39.6)	
Recurrence				0.097
No	300 (83.3)	226 (75.3)	74 (24.7)	
Yes	60 (16.7)	39 (65.0)	21 (35.0)	
Metastatic events				0.019*
No	310 (86.1)	235 (75.8)	75 (24.2)	
Yes	50 (13.9)	30 (60.0)	20 (40.0)	
Lung				1.000
No	344 (95.6)	253 (73.5)	91 (26.5)	
Yes	16 (4.4)	12 (75.0)	4 (25.0)	
Bone				0.210
No	339 (94.2)	252 (74.3)	87 (25.7)	
Yes	21 (5.8)	13 (61.9)	8 (38.1)	
Brain				0.697
No	354 (98.3)	261 (73.7)	93 (26.3)	
Yes	6 (1.7)	4 (66.7)	2 (33.3)	
Liver				0.011*
No	348 (96.7)	260 (74.7)	88 (25.3)	
Yes	12 (3.3)	5 (41.7)	7 (58.3)	
Contralateral supraclavicular lymph node metastasis				0.447
No	358 (99.4)	264 (73.7)	94 (26.3)	
Yes	2 (0.6)	1 (50.0)	1 (50.0)	
Death				0.190
No	320 (88.9)	239 (74.7)	81 (25.3)	
Yes	40 (11.1)	26 (65.0)	14 (35.0)	
Chemotherapy				0.919
No	10 (2.7)	8 (80.0)	2 (20.0)	
Yes	350 (97.2)	257 (73.4)	93 (26.6)	0.644
Anthracycline and taxane-based regimens	214 (59.4)^a^	159 (74.3)	55 (25.7)	
Others	136 (37.7)^b^	98 (72.1)	38 (27.9)	
Bone				0.210
No	339 (94.2)	252 (74.3)	87 (25.7)	
Yes	21 (5.8)	13 (61.9)	8 (38.1)	
Brain				0.697
No	354 (98.3)	261 (73.7)	93 (26.3)	
Yes	6 (1.7)	4 (66.7)	2 (33.3)	
Liver				0.011*
No	348 (96.7)	260 (74.7)	88 (25.3)	
Yes	12 (3.3)	5 (41.7)	7 (58.3)	

Data are expressed as the patient number (%) or mean ± SD. Statistically significant differences were defined as *P* < 0.05.

*LNM* lymph node metastasis, *IDC* invasive ductal carcinoma, *IM* immunomodulatory subtype, *LAR* luminal androgen receptor subtype, *BLIS* basal-like immune-suppressed subtype, *MES* mesenchymal-like subtype.

^a^Includes 25 cases who received additional platinum regimens.

^b^Consists of the following: single-agent taxane (*n* = 22), single-agent anthracycline (*n* = 39), single-agent platinum (*n* = 2), combination of taxane and platinum (*n* = 40), and unknown agents (*n* = 33).

### The expression level of GPER was correlated with the immunohistochemistry (IHC) score in TNBC tissues

**Fig. 1:**
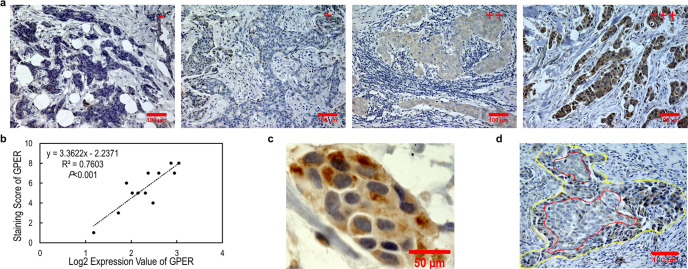
Immunohistochemical staining for GPER. **a** Immunohistochemical staining for GPER with different intensity. The staining is graded as negative (−), weak (+), moderate (++), and strong (+++). Scale bar, 100 μm. **b** Scatter plots show that the IHC score is linearly correlated with the log2 expression value of GPER in the results of Pearson correlation coefficient calculation, which is statistically significant. Pearson correlation coefficient *R*^2^ and *p*-value are given in the scatter plot. **c** Image showing IHC staining of GPER is only observed in cytoplasmic. Scale bar, 50 μm. **d** Representative image of weak GPER staining in the core area and strong GPER staining in the corresponding margin. Scale bar, 100 μm.

In our cohort, GPER expression at the mRNA level, shown as log2 (FPKM + 1) expression value, was of normal distribution in TNBC tissues (Supplementary Fig. 1). To verify the expression of GPER detected by RNA sequencing, samples from 12 patients were randomly selected for IHC staining using an antibody against GPER. As expected, we found varying staining intensities of GPER in these tissues (Fig. 1a) and the IHC scoring was significantly correlated with the log2 expression value of GPER (Fig. 1b). Although both cytoplasmic and membrane staining of GPER were reported, we observed, even by oil lens, only cytoplasmic patterns in these samples (Fig. 1c). Interestingly, we observed heterogeneity in GPER staining. In some nests, weak staining was localized in the core area while strong staining was observed at the margin (Fig. 1d).

### Association between GPER and clinicopathological variables of TNBC

According to the cutoff value of RNA sequencing results, low and high GPER expression levels were detected in 73.6% (265/360) and 26.4% (95/360) of patients, respectively (Table 1). The association of GPER expression with clinicopathological variables was assessed. High GPER expression was significantly correlated with necrosis in the cancer nest (*P* = 0.027) and mRNA subtype (*P* = 0.001) of TNBC according to our classification^36^. Compared to the GPER-low group, the GPER-high group demonstrated increased incidences of total metastatic events (21.1% vs. 11.3%; *P* = 0.019) and liver metastasis (7.4% vs. 1.9%; *P* = 0.011) in the follow-up. Other clinicopathological variables, such as age, menopausal status, and tumor size, had no significant correlations with GPER expression.

### The distribution of GPER is different among subtypes of TNBC

**Fig. 2:**
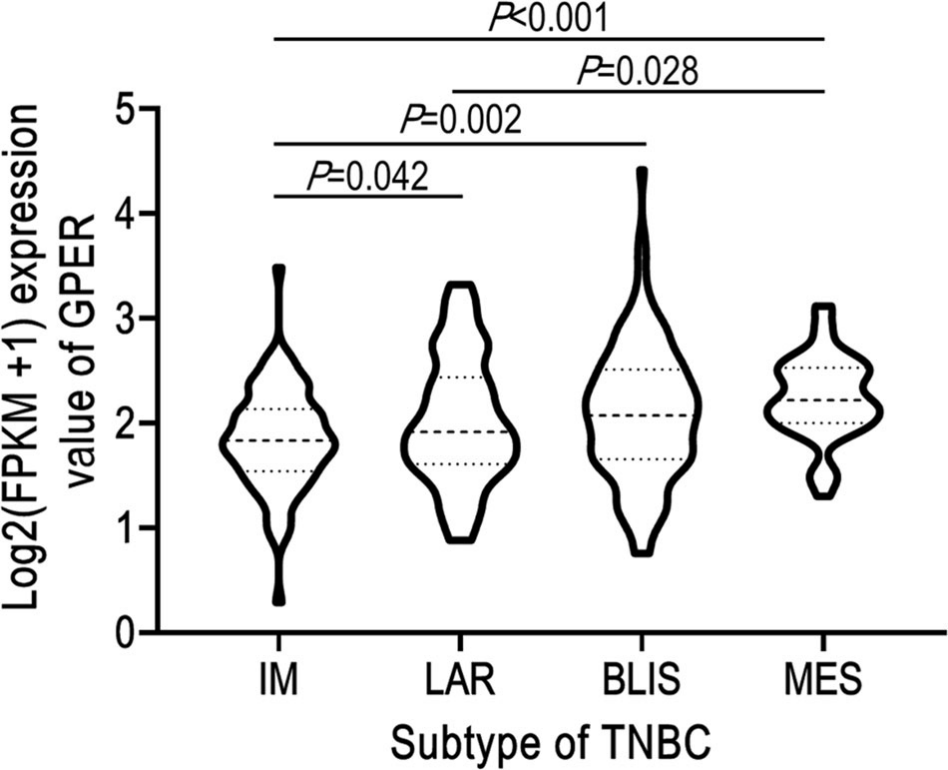
Violin diagram showing the distribution of GPER expression among subtypes of TNBC. Statistically significant differences were defined as *P* < 0.05. Abbreviations: IM immunomodulatory subtype; LAR luminal androgen receptor subtype; BILS basal-like immune-suppressed subtype; MES mesenchymal-like subtype.

As mentioned, tumors in this cohort were classified into four transcriptome-based subtypes and the distribution of GPER expression was significantly different among subtypes of TNBC (Table 1). Thus, we quantitatively compared the abundance of GPER among subtypes of TNBC by ANOVA analysis. Intriguingly, the abundance of GPER was the lowest in the IM subtype which presented the best prognosis. Respectively, GPER abundance was highest in the MES subtype which presented the worst prognosis among all TNBC subtypes (Fig. 2). A significant difference was found between LAR and IM (*P* = 0.042), BILS and IM (*P* = 0.002), MES and IM (*P* < 0.001) as well as MES and LAR (*P* = 0.028). This may suggest that GPER is correlated with a higher risk subtype of TNBC.

### High expression of GPER predicted worse DMFS in TNBC patients

**Fig. 3:**
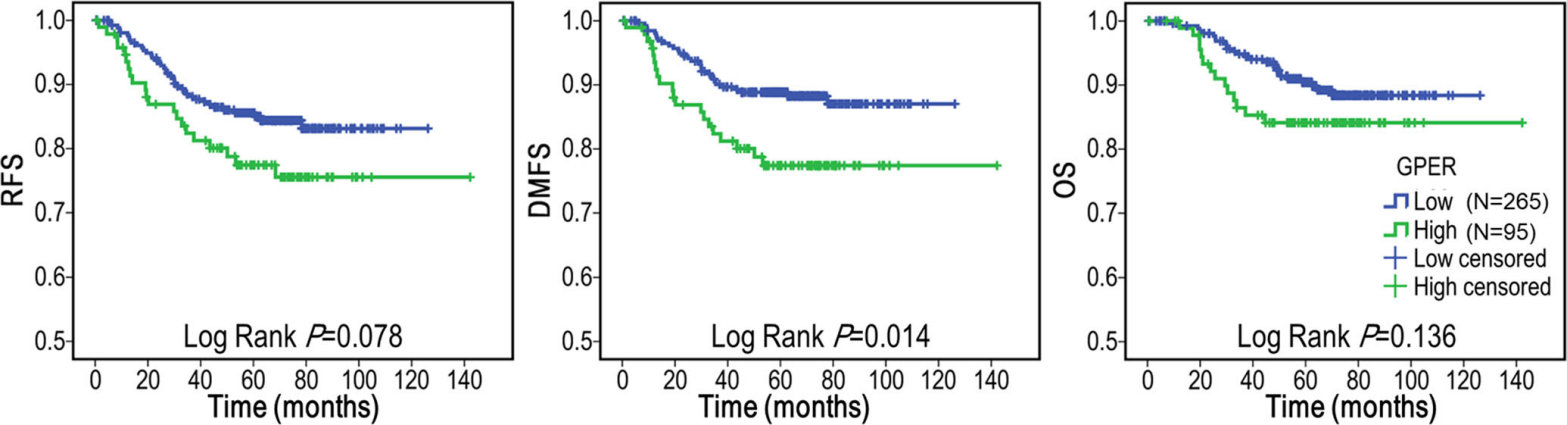
Kaplan–Meier survival curves of RFS, DMFS, and OS between GPER-low and GPER-high groups in patients with TNBC. Statistically significant differences were defined as *P* < 0.05. Abbreviations: RFS relapse-free survival; DMFS distant metastasis-free survival; OS overall survival.

Survival outcomes were analyzed to explore the potential of GPER as a survival predictor. Kaplan–Meier analysis of this cohort revealed a trend towards reduced RFS (Log Rank *P* = 0.078), DMFS (Log Rank *P* = 0.014), and OS (Log Rank *P* = 0.136) in patients with high GPER expression TNBCs. Note, that statistical significance is only presented in DMFS (Fig. 3).

Additionally, a Cox proportional hazard regression model was used to identify biomarkers and clinicopathological factors affecting the prognosis of patients with TNBC (Table 2). The univariate analysis confirmed that GPER was a significant prognostic factor for DMFS (hazard ratios (HR) = 2.01; 95% confidence interval (CI), 1.14–3.54; *P* = 0.016) in TNBC patients. However, the further multivariate analysis failed to provide evidence for GPER (HR = 1.642; 95% CI, 0.92–2.92; *P* = 0.091) as an independent prognostic factor. Additionally, the prognostic value of GPER was not significant in RFS and OS neither by univariate nor multivariate analysis. Referring to the other clinicopathological variables, LNM status, and TNM stage were identified as prognostic indicators for RFS, DMFS, and OS in the univariate model, while mRNA subtype was only associated with DMFS. Multivariate analysis proved that TNM stage was a significant independent prognostic factor for RFS, DMFS, and OS in TNBC patients. Besides, LNM status was also suggested as an independent prognostic factor for RFS and DMFS, but not OS.

**Table 2:** Univariate and multivariate survival analyses of clinicopathological factors and GPER expression.

		Univariate model				Multivariate model			
		*P*	HR	*β*	95% CI for HR	*P*	HR	*β*	95% CI for HR
RFS	Age (<50 vs. >=50)	0.106	0.658	−0.418	0.396–1.093				
	Menopause status (pre-menopause vs. post-menopause)	0.547	0.853	−0.159	0.509–1.430				
	Nuclear grade (G2 vs. G3)	0.109	0.633	−0.457	0.362–1.107				
	Tumor size (pT1 vs. pT2/3)	0.332	1.309	0.270	0.760–2.256				
	LNM (positive vs. negative)	<0.001	3.474	1.245	2.015–5.987	0.018	2.185	0.782	1.147–4.165
	TNM stage (III vs. I/II)	<0.001	4.763	1.561	2.828–8.022	0.001	2.963	1.086	1.599–5.493
	Necrosis (positive vs. negative)	0.434	1.270	0.239	0.698–2.309				
	mRNA subtype (IM vs. others)	0.088	0.540	−0.616	0.266–1.097				
	Intrinsic subtype (Basal vs. others)	0.565	1.183	0.168	0.667–2.096				
	Histology (IDC vs. others)^a^	-	-	-	-				
	Ki67 (>=30% vs. <30%)	0.425	1.305	0.266	0.678–2.514				
	GPER (high vs. low)	0.081	1.604	0.473	0.944–2.727				
DMFS	Age (<50 vs. >=50)	0.178	0.682	−0.383	0.391–1.190				
	Menopause status (pre-menopause vs. post-menopause)	0.388	0.781	−0.247	0.445–1.370				
	Nuclear grade (G2 vs. G3)	0.392	0.760	−0.275	0.405–1.425				
	Tumor size (pT1 vs. pT2/3)	0.395	1.294	0.258	0.714–2.344				
	LNM (positive vs. negative)	<0.001	5.020	1.613	2.622–9.610	0.005	3.005	1.100	1.403–6.432
	TNM stage (III vs. I/II)	<0.001	6.490	1.870	3.717–11.334	0.001	3.132	1.142	1.622–6.046
	Necrosis (positive vs. negative)	0.116	1.721	0.543	0.874–3.388				
	mRNA subtype (IM vs. others)	0.022	0.340	−1.080	0.135–0.856	0.082	0.432	−0.840	0.167–1.113
	Intrinsic subtype (Basal vs. others)	0.684	1.140	0.131	0.606–2.145				
	Histology (IDC vs. others)	0.993	0.995	−0.005	0.358–2.765				
	Ki67 (>=30% vs. <30%)	0.271	1.530	0.425	0.717–3.264				
	GPER (high vs. low)	0.016	2.009	0.698	1.141–3.538	0.091	1.642	0.496	0.923–2.920
OS	Age (<50 vs. >=50)	0.489	0.802	−0.221	0.428–1.501				
	Menopause status (pre-menopause vs. post-menopause)	0.828	1.075	0.072	0.561–2.058				
	Nuclear grade (G2 vs. G3)	0.804	0.913	−0.091	0.447–1.866				
	Tumor size (pT1 vs. pT2/3)	0.775	1.100	0.095	0.574–2.106				
	LNM (positive vs. negative)	0.001	3.225	1.171	1.663–6.252	0.197	1.720	0.542	0.754–3.924
	TNM stage (III vs. I/II)	<0.001	5.445	1.695	2.907–10.200	0.001	3.871	1.354	1.772–8.457
	Necrosis (positive vs. negative)	0.192	1.658	0.506	0.776–3.543				
	mRNA subtype (IM vs. others)	0.204	0.570	−0.562	0.239–1.358				
	Intrinsic subtype (Basal vs. others)	0.569	0.798	−0.225	0.368–1.732				
	Histology (IDC vs. others)	0.500	1.632	0.490	0.394–6.764				
	Ki67 (>=30% vs. <30%)	0.780	1.117	0.111	0.515–2.425				
	GPER (high vs. low)^a^	-	-	-	-				

Covariates with *P* < 0.05 in the univariate model were included in the further multivariate model. Statistically significant differences were defined as *P* < 0.05.

^a^Covariates that did not satisfy the proportional hazards assumption with Schoenfeld residuals test.

### High GPER expression predicted worse survival in high-risk TNBC patients

**Fig. 4:**
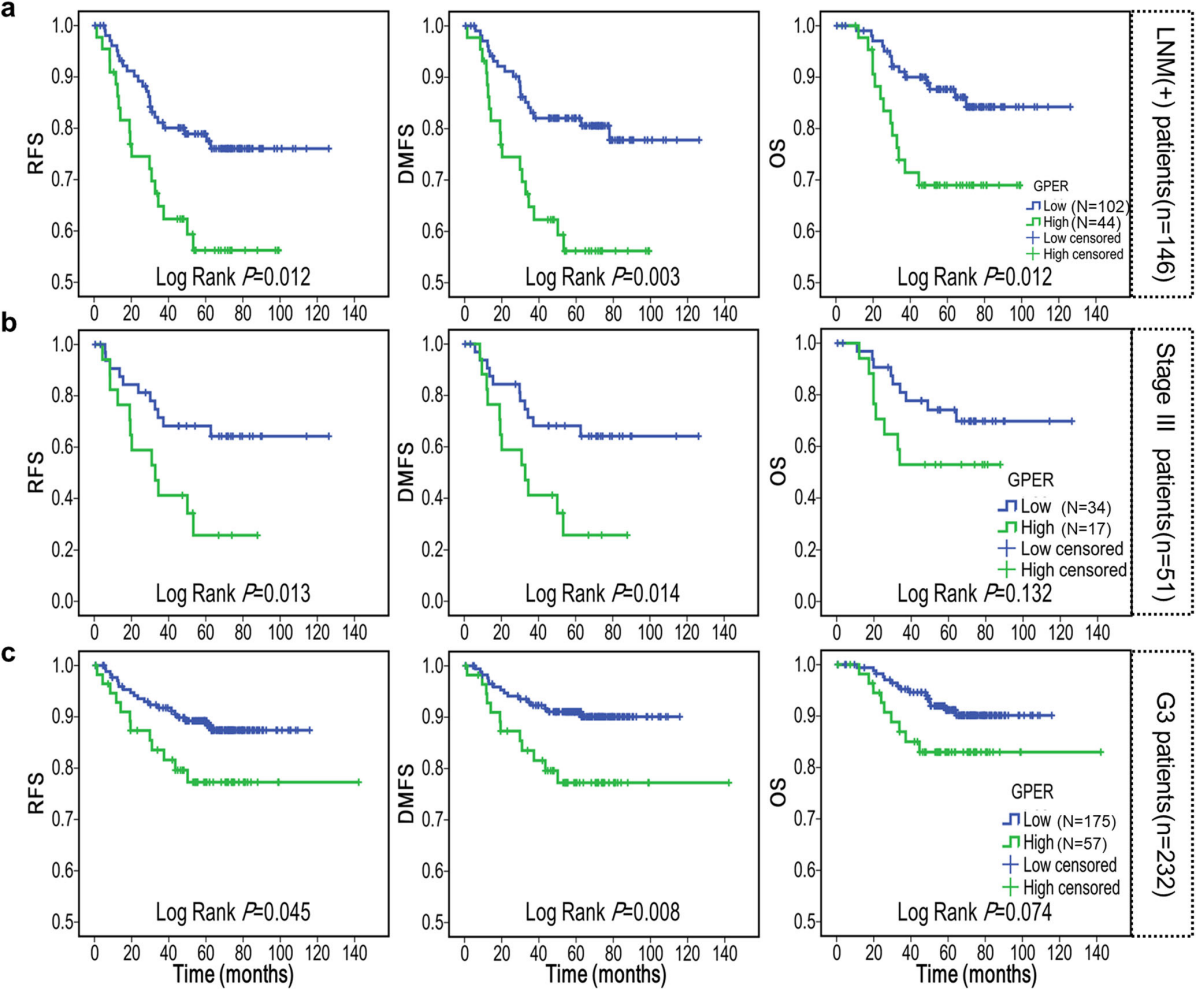
Kaplan–Meier curves between GPER-low and GPER-high groups in high-risk patients. **a** Kaplan–Meier curves of RFS, DMFS and OS in LNM (+) patients. **b** Kaplan–Meier curves of RFS, DMFS and OS in stage III patients. **c** Kaplan–Meier curves of RFS, DMFS and OS in patients with G3 tumors. Statistically significant differences were defined as *P* < 0.05.

Intriguingly, the prognostic value of GPER, not only for DMFS but also for RFS and OS, dramatically increased when stratifying for known risk factors. Revealed by Kaplan–Meier analysis, high expression of GPER was linked significantly to the worse RFS (Log Rank *P* = 0.012), DMFS (Log Rank *P* = 0.003), and OS (Log Rank *P* = 0.012) in LNM (+) patients while no difference was found in LNM (−) patients (Fig. 4a and Supplementary Fig. 2). Referring to stage III patients, high GPER expression correlated with lower RFS (Log Rank *P* = 0.013) and DMFS (Log Rank *P* = 0.014), and a trend toward correlating with OS (Log Rank *P* = 0.132) (Fig. 4b and Supplementary Fig. 2). Similarly, high GPER expression is also associated with reduced RFS (Log Rank *P* = 0.045) and DMFS (Log Rank *P* = 0.008), and a trend toward OS (Log Rank *P* = 0.074) in patients with G3 tumors (Fig. 4c and Supplementary Fig. 2). Thus, the predictive value of GPER seems to be increased in TNBC patients with additional risk factors, including LNM positivity, higher nuclear grade and later TNM stage.

### GPER was linked to pro-metastatic pathways in the transcriptomic landscape of Chinese TNBC patients

**Fig. 5:**
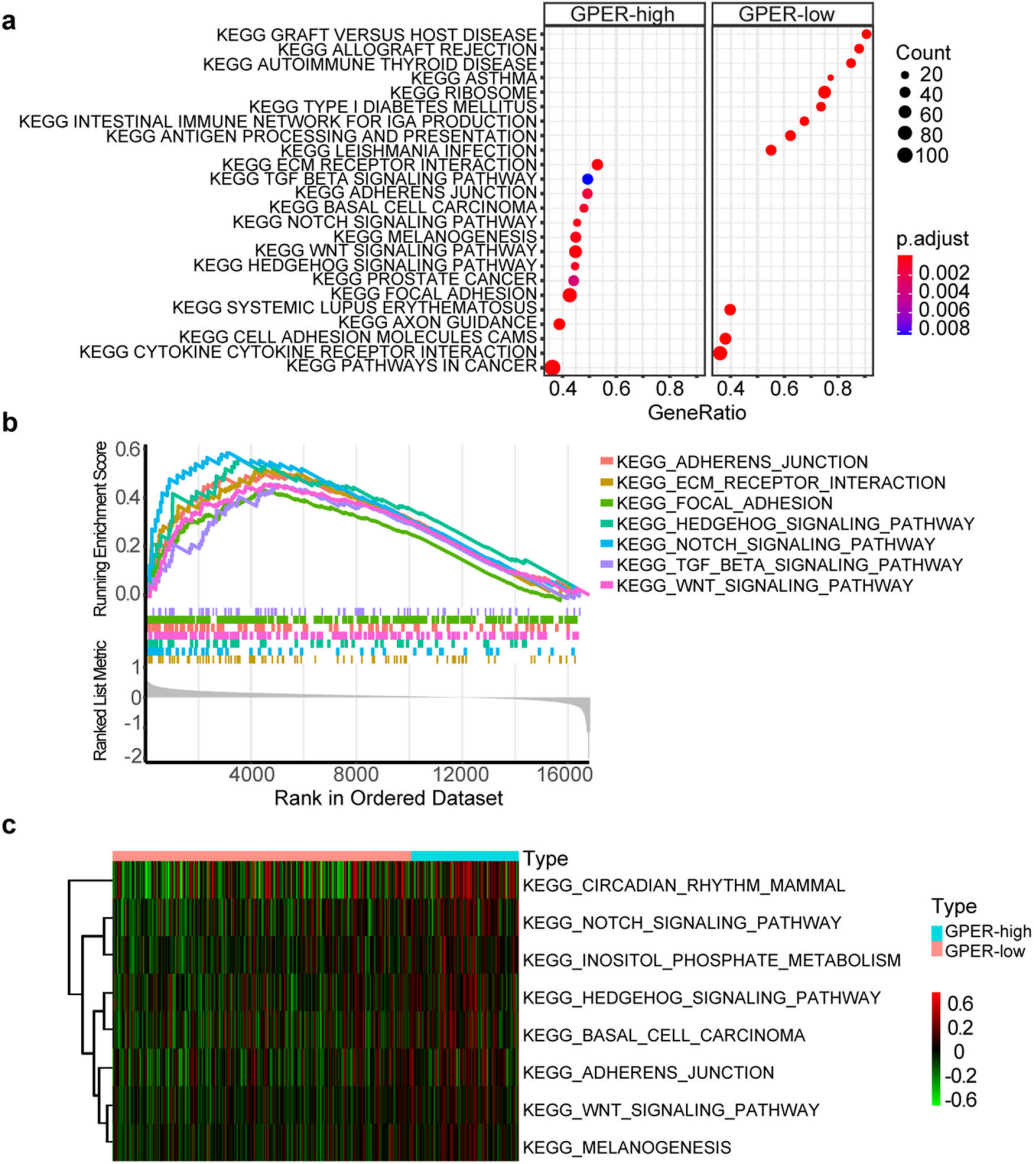
The GSEA and GSVA results of GPER correlated pathways based on the transcriptomic data of 360 TNBC patients. **a** Dotplot showing the twelve most significantly upregulated pathways in GPER-high and GPER-low groups from GSEA results. In the group of high GPER expression, seven of the twelve upregulated pathways are correlated with promoting tumor metastasis. **b** GSEA-plots showing the upregulated pro-metastatic pathways in the GPER-high group. **c** Heatmap for the eight most significantly upregulated pathways in GPER-high group by GSVA. Statistically significant differences were defined as adjusted *P*-value < 0.05.

To better understand the estrogenic carcinogenesis mediated by GPER in TNBC, we applied bioinformatics analysis based on the transcriptomic profiles of 360 TNBC patients in our cohort. The gene set enrichment analysis (GSEA) and gene set variation analysis (GSVA) were performed between the GPER-high and GPER-low groups of TNBC patients. Based on the results of GSEA analysis, the dot plot shows the significantly enriched GPER-related pathways (Fig. 5a). Of note, the enriched pathways with pro-metastatic characteristics included Focal adhesion, WNT signaling pathway, ECM receptor interaction, NOTCH signaling pathway, Hedgehog signaling pathway, Adherens junction pathway, and TGF beta signaling pathway, as indicated by their respective adjusted p-values and GSEA-plots (Fig. 5a, b). Additionally, the GSVA analysis was conducted using KEGG gene sets. Firstly, 186 KEGG pathways were quantified using the GSVA package. Then, differential analysis was conducted to find specific pathways for GPER-high and GPER-low groups. Similar to the GSEA results, the GSVA results also showed that the pro-metastasis pathways that are significantly enriched in the GPER-high group include NOTCH signaling pathway, Hedgehog signaling pathway, WNT signaling pathway, and Adherens junction pathway (Fig. 5c).

## Discussion

TNBC has the worst prognosis of all breast cancer subtypes and the lack of well-defined molecular targets is the main challenge to treat TNBC patients^1,2^. Although estrogens largely contribute to the development and progression of breast cancer, estrogen carcinogenesis was long disregarded in TNBC. In the present study, we revealed that GPER expression was associated with the aggressive subtype of TNBC. High GPER expression predicted reduced DMFS in our cohort. Especially in high-risk patients with G3 tumors, LNM (+) or stage III, the prognostic significance was increased. Transcriptome-based bioinformatics analysis revealed that GPER was linked to pro-metastatic pathways in the Chinese cohort of TNBC.

Theoretically, GPER, as a membrane receptor belonging to the GPCR superfamily, is significantly different from ER-α, a nuclear steroid hormone receptor. In biology, the differences between GPER and ER-α include their subcellular distribution, structure, affinity to E2, ligands pattern, the process and effects in response to E2. These differences have attracted a surge of interest and are well-reviewed in the literature^12,13,24^. Meanwhile, GPER is not totally accepted as a cognate ER in related debates^24–27^. GPER was reported to coimmunoprecipitate with ER-α in MCF-7 cells and to repeatedly correlate with ER-α positivity in primary breast cancers^30,32,37^, thus it may contribute to estrogenic responses as a collaborator. GPER didn’t even respond to stimulation of E2 and G1 in some cell models^38^. However, GPER was detected in tissues and cell lines of not only breast cancer but also other organs lacking ER-α expression^12,13^. Furthermore, GPER was observed to bind E2 directly in several cell lines lacking ER-α^10,39,40^. In clinical series, inverse correlation or non-significant association was also found between GPER and ER-α^41^. Due to the lack of ER-α, it was assumed that TNBC is estrogen-insensitive. However, increasing circulating estrogen levels were sufficient to promote the formation and progression of ER-α negative cancers including TNBC and pharmacological inhibition of estrogen synthesis after pregnancy prevented the formation of ER-α negative tumors^42^. Furthermore, we found that the mRNA expression level of GPER, which is positively correlated with the staining score of the GPER protein (*R*² = 0.7603), is widely distributed in this large Chinese cohort of TNBC tissues, in line with earlier detection of GPER in tissues and cell lines of TNBC^15–20^. Taken together, these results indicate a functional role of GPER as an alternative ER and the potential as a mediator of estrogen carcinogenesis in TNBC. However, further identification, especially based on GPER protein expression, is needed.

There are some disputes about the subcellular localization of GPER. Known as a GPCR, GPER can be detected on the cell surface and is involved in signal transduction events, such as Ca^2+^ mobilization^10^, NO generation^43^, ERK activation^44^, and growth factor release^45^. GPER was also observed to locate in the endoplasmic reticulum and to act as an endoplasmic reticulum stressor that induces growth inhibition^46^ and apoptotic cell death^47^. GPER distribution in mitochondria, Golgi apparatus, and nucleus also reported^48^. The different subcellular locations of the GPER may have different biological implications. Importantly, our group has found that GPER exists in the nucleus and is translocated from the nucleus to the cytoplasm under E2 stimulation^49^. In this study, GPER staining only exhibited in the cytoplasm may be due to the small sample size, different functional status of cells, and less sensitive detection methods.

Physiologically, the expression pattern of GPER is likely to be tissue-dependent and developmentally regulated. In the mammary ductal epithelia, GPER abundance was varied with the estrous cycle^50^. Yet, the trend of GPER expression in breast cancer development and progression remains elusive. In an early study, GPER was detected in every single breast sample from 12 healthy donors^30^. The GPER expression in normal breast tissue was at a medium level in the Human Protein Atlas database^51^. In comparison, GPER expression was reported to decrease in tumor tissues^52^, while inflammatory breast cancer, an aggressive type of breast cancer, exhibited stronger intensity in staining against GPER^41^. Interestingly, GPER expression was correlated with the tumor subtype in a large cohort and strong staining was significantly more prevalent among TNBCs^33^. Notably, when TNBC subtypes were classified by transcriptomic profile, the expression of GPER correlated with the subtype, and the highest level was found in the MES subtype, which presented the worst prognosis in all TNBC subtypes. In addition, weak positivity was localized in the core area while strong positivity was observed at the margin in some nests by IHC staining, implying that GPER abundance may be increased during the invasion of cancer cells. It also implied a link between GPER and the aggressiveness of the breast cancer, that is, high expression of GPER was associated with more frequent necrosis in tumor sections. Besides, metastatic or recurrent cancer tumors also showed higher levels of GPER expression than corresponding primary tumor^19,31,53^. Meanwhile, the aggressive cell lines of uterine and ovarian cancer (JEG and Hec50) expressed a much higher level of GPER than their associated normal cell lines (HTR8 and H, respectively)^10^. In general, GPER expression likely increases with the development and progression while it indicates the natural aggressiveness of breast cancer.

The prognostic value of GPER for breast cancer patients has been addressed in multiple studies^30–34^. However, controversial findings have been reported, even in meta-analysis^54^. The biological function of GPER largely depends on the cellular background in vitro^12,13^, thus the prognostic role of GPER should be evaluated in a specific subtype of breast cancer. In this respect, the contribution of GPER should be genuine in TNBC. In this large cohort of patients, high GPER expression was linked to poor DMFS and exhibited prognostic significance, especially in high-risk patients with G3 tumors, LNM (+) and stage III. These results were consistent with earlier reports as follows: In an earlier report with a small cohort of TNBC (*n* = 18), GPER was thought to have a poorer prognosis with a trend towards increased recurrences^35^. High GPER expression was also related to decreased outcomes, including the local RFS, DDFS, OS, and PFS in a Chinese cohort (*n* = 249)^17^. In a retrospective TNBC study (*n* = 199), GPER and estrogen-related receptor α (ERR-α) synergistically predicted poor patient outcomes^55^. In summary, studies seem to agree that GPER can mediate estrogen carcinogenesis and promote the progression of TNBC. Hence, GPER could be of significant prognostic value. However, a larger cohort and longer follow-up are needed to address this issue.

As aforementioned, early metastasis is underlying the poor prognosis of patients with TNBC^2,3^. Interestingly, GPER was linked to the metastatic behaviors of TNBC cells in vitro and in vivo^16,18,19,21,56^. Ligands including E2 and bisphenol A were claimed to trigger GPER to promote migration and metastasis of TNBC cells^21–23^. E2 also induced the up-regulation of estrogen-related receptor α expression via GPER activation, enhanced the migration and invasion of TNBC cells^55^. Increased GPER expression was observed at the invasive margin in some nests, implying that GPER activation may contribute to the invasion of cancer cells. A recent bioinformatics analysis showed that GPER was correlated with pro-metastatic pathways in ER-α negative breast cancer by Maggiolini et al.^28^. We conducted a similar analysis in our large single-center study and, as expected, GPER was correlated with multiple pro-metastatic pathways. Among these pathways, the Focal adhesion and ECM receptor interaction pathways, both of which were deeply involved in cancer invasion and metastasis, were also the most significant GPER-related pro-metastatic pathways in the aforementioned study. Actually, estrogenic GPER signaling was ascertained to trigger focal adhesion kinase phosphorylation to increase focal adhesion points and cellular migration in TNBC cells^21^. Other pro-metastatic pathways including NOTCH signaling, Hedgehog signaling, WNT signaling, and Adherens junction were also correlated with GPER in our analysis. However, the GPER-related pro-metastatic pathways identified by Maggiolini et al. were somewhat different with those in this study, maybe due to the differences of tumor subtype (ER-α negative vs. TNBC) and race of patients (mixed vs. Chinese). To our knowledge, performing the analysis in homogeneous TNBC cohort is significant since heterogeneity of TNBC is high enough and excluding the effect of crosstalk between estrogenic GPER signaling and HER2 signaling is necessary. Anyway, given that GPER mediates the transcriptional regulation of estrogen in TNBC cells and other breast cancer cell lines^19,57^, GPER may trigger variable signal transduction events to enhance the multi-steps of metastasis. However, to better understand the functional role of GPER, the mechanisms by which GPER contributes to the progression of TNBC need further clarification. Accordingly, new endocrine therapy by blocking GPER-related signaling may be an effective strategy for TNBC. Since several antagonists of GPER have been synthesized, employing them as endocrine therapy agents is accessible, while more basic and translational research is needed to confirm this potency.

In summary, in a unique and large Chinese cohort of TNBC with long-term follow-up, we evaluated the expression of GPER and showed that GPER expression correlates with the subtype of TNBC, with a trend to increase the aggressiveness of tumors. We concluded that GPER has significant prognostic value in TNBC and is significantly linked to the worse survival, especially in high-risk patients with LNM (+), G3 or stage III tumors. Furthermore, bioinformatics analysis was performed based on the transcriptomic profile of the TNBC cohort and the correlation between high GPER expression and pro-metastatic pathways was verified, suggesting that GPER has significant functional roles in TNBC metastasis. Taken together, this may provide evidence that GPER mediates metastatic estrogen carcinogenesis in TNBC. Considering the great urgency for clinicians and researchers to develop efficient molecular targets and biomarkers, GPER could be a promising candidate for TNBC therapy and diagnosis.

## Methods

### Patient recruitment

From January 1, 2007 to December 31, 2014, primary tumor tissue and blood samples were obtained from 504 consecutive female Chinese patients with TNBC treated at Fudan University Shanghai Cancer Center (FUSCC). Among these patients, 279 had whole exome sequencing (WES) data on primary tumor tissue and paired blood samples, 401 had copy-number alteration (CNA) data and 360 had RNA sequencing data on primary tumor tissue. 360 patients with RNA sequencing data were enrolled in this study according to the following defined criteria: (1) female patients diagnosed with unilateral disease; (2) histologically confirmed the ER-α (−), PR (−), and HER2 (−) phenotype; (3) no evidence of distant metastasis at diagnosis; (4) sufficient frozen tissues available for further research. The examination results for chest computed tomography (CT), bone scans, abdominal ultrasound, bilateral mammography, breast ultrasound, and/or magnetic resonance imaging (MRI) were collected to ascertain no metastasis beyond breasts and axillary lymph nodes metastasis before the surgery. Ethical review and approval were waived for this study, due to the data reported in this paper have been described in our published article^36^ and deposited in the NCBI Sequence Read Archive (SRA: SRP157974). All patients provided written informed consent for data and tissue use.

### RNA sequencing and transcriptomic profiling

RNA sequencing data and transcriptomic profiling of 360 patients with TNBC from FUSCC were used in the current study. Detailed sample preparation, library preparation, sequencing, and raw data processing were described in our earlier publication^36^. The RNA sequencing data have been deposited in NCBI Sequence Read Archive, with accession number SRP157974.

Four stable clusters, IM, LAR, MES, and BLIS were identified after analyzing the robustness of the classification using k-means clustering with the details available in our previous study^36^. Our classification system, named FUSCC, correlated well with the Lehmann/Pietenpol classification system.

### Clinicopathological data

The ER-α, PR, and HER2 status of the breast tumor samples were confirmed by two experienced pathologists based on immunochemical analysis and in situ hybridization. ER-α and PR status were classified as negative using a cutoff of 1%, according to the American Society of Clinical Oncology/College of American Pathologists (ASCO/CAP) guidelines^58^. HER2 status was defined as negative with 0, 1+ as well as 2+ on immunohistochemistry without HER2 gene amplification on fluorescence in situ hybridization (FISH)^59^. TNBC was defined as ER-α, PR, and HER2 negative in accordance with the St. Gallen International Expert Consensus^60^. Clinicopathological features, including age at diagnosis, menopausal status, tumor histologic type, tumor size, LNM, histologic grade, TNM stage and ER-α, PR, HER2, and Ki67 status, were analyzed. The tumor stage based on the TNM stage was assessed according to the criteria established by the 8th edition American Joint Committee on Cancer (AJCC 8th) staging manual of breast cancer.

### Patient follow-up

Follow-up of all patients in this cohort was completed on June 11, 2019. The median length of follow-up was 67.1 months with an interquartile range of 53.9–79.9 months. RFS was defined as the time from diagnosis to first recurrence or a diagnosis of contralateral breast cancer. DMFS was defined as the time from diagnosis to first distant metastasis. OS was defined as the time from diagnosis to death. Patients without events were censored from the time point of the last follow-up.

### Immunohistochemistry staining and scoring

Immunohistochemistry staining was performed using an SP900 Kit (Zhongshan Golden Bridge) according to the manufacturer’s protocol. Briefly, deparaffinized tissue sections of 4 μm thickness were heated for antigen retrieval at 95 °C for 15 min in 10 mM citric acid buffer (pH 6.0). After treatment with 3% H_2_O_2_ for 10 min to quench endogenous peroxidase activity, the sections were blocked using goat serum and then incubated with the primary antibody targeting GPER (1:250, ab39742, Abcam, USA) at a 1:200 dilution at 4 °C for 16 h. The section treated with PBS worked as a negative control. Following treatment of horseradish peroxidase-conjugated goat anti-rabbit IgG for 30 min at 37 °C, sections were developed using diaminobenzidine (DAB) (Zhongshan Golden Bridge) and nuclei were counterstained with Mayer’s modified hematoxylin. As indicated by Filardo et al.^30^, reduction mammoplasty tissue was used as a positive control.

Two observers microscopically evaluated the intensity, extent and subcellular distribution of GPER using a modified semi-quantitative scoring system. GPER scores were assigned as follows: the percentage of positive cells was categorized as 0 (negative staining in all cells), 1 (<1% cells stained), 2 (1–10% of cells stained), 3 (11–30% cells stained), 4 (31–70% cells stained) or 5 (71–100% cells stained), and staining intensity was categorized as 0 (negative), 1 (weak), 2 (moderate) and 3 (strong). Adding the two scores together yielded a maximum score of 8. The final scores were grouped into GPER negative (score 0−4) and positive (score 5−8) categories for statistical analyses to reduce inter-observer differences.

### Bioinformatics analysis

The 360 patients with TNBC were divided into 26.4% (95/360) patients with high GPER expression and 73.6% (265/360) patients with low GPER expression. RNA sequencing data of 360 TNBC patients were used to determine the differences between groups through the R software limma package^61^ and GSEA was applied to conduct gene enrichment analysis by using clusterProfiler package^62^. GSVA analysis was performed on log2 (FPKM + 1) expression values by using GSVA package^63^. The “c2.cp.kegg.v7.4.entrez.gmt” and “c2.cp.kegg.v7.2.symbols.gmt” gene sets were downloaded from the Molecular Signatures Database^64^.

### Statistical analysis

All statistical analyses were done by using the SPSS standard version 25 software and Stata version 13.0 software. For the division of high and low expression groups of GPER, the X-tile software was used to generate the optimal cut-off value^65^. Continuous quantitative data were expressed as mean ± standard deviation (SD) and categorical qualitative data as percentage. Data from the two patient groups were statistically compared using the chi-square test or t test as appropriate. The Pearson correlation coefficient was used to analyze the correlation between IHC scoring and the log2 expression value of GPER. ANOVA test was used to determine the differences of GPER expression among TNBC subtypes. RFS, DMFS, and OS curves were drawn using the Kaplan–Meier methods and were compared using Log-rank tests. Univariate and multivariate analyses of the patients’ survival were performed using the Cox proportional hazards regression model and the HRs with 95% CIs were calculated. Statistics of *P* < 0.05 in univariate analysis were used as inclusion criteria for covariates in the final multivariate model. The proportional hazards assumption was tested by using Schoenfeld residual tests. If the assumption of proportional hazards was not valid, time-dependent covariates were introduced. All tests were two-sided and *P* < 0.05 was deemed statistically significant.

### Reporting summary

Further information on research design is available in the Nature Research Reporting Summary linked to this article.

## Supplementary information

Supplementary Material

Reporting Summary

## Data Availability

RNA sequencing data that support the findings of this study have been deposited in NCBI Sequence Read Archive with the accession codes SRP157974. All other relevant data are available from the corresponding author on request.

## Code availability

The code used to process and analyze the RNA sequencing data is available from the corresponding author upon reasonable request.
