# Supplementary material for: High GPER expression in triple-negative breast cancer is linked to pro-metastatic pathways and predicts poor patient outcomes
Source: NPJ Breast Cancer. 2022 Aug 30;8:100. doi: 10.1038/s41523-022-00472-4 (PMC9427744; doi:10.1038/s41523-022-00472-4)
Supplement: Supplementary file 1 — Supplementary Material [file 41523_2022_472_MOESM1_ESM.pdf]

Supplementary Figures

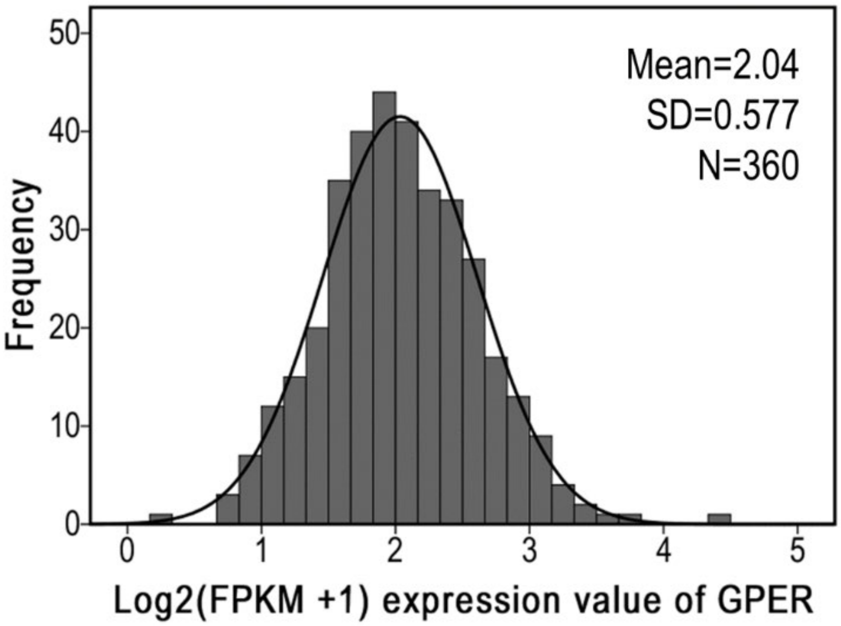

Supplementary Figure 1. The log2 (FPKM+1) expression value of GPER at mRNA level met the normal distribution in 360 TNBC tissues.

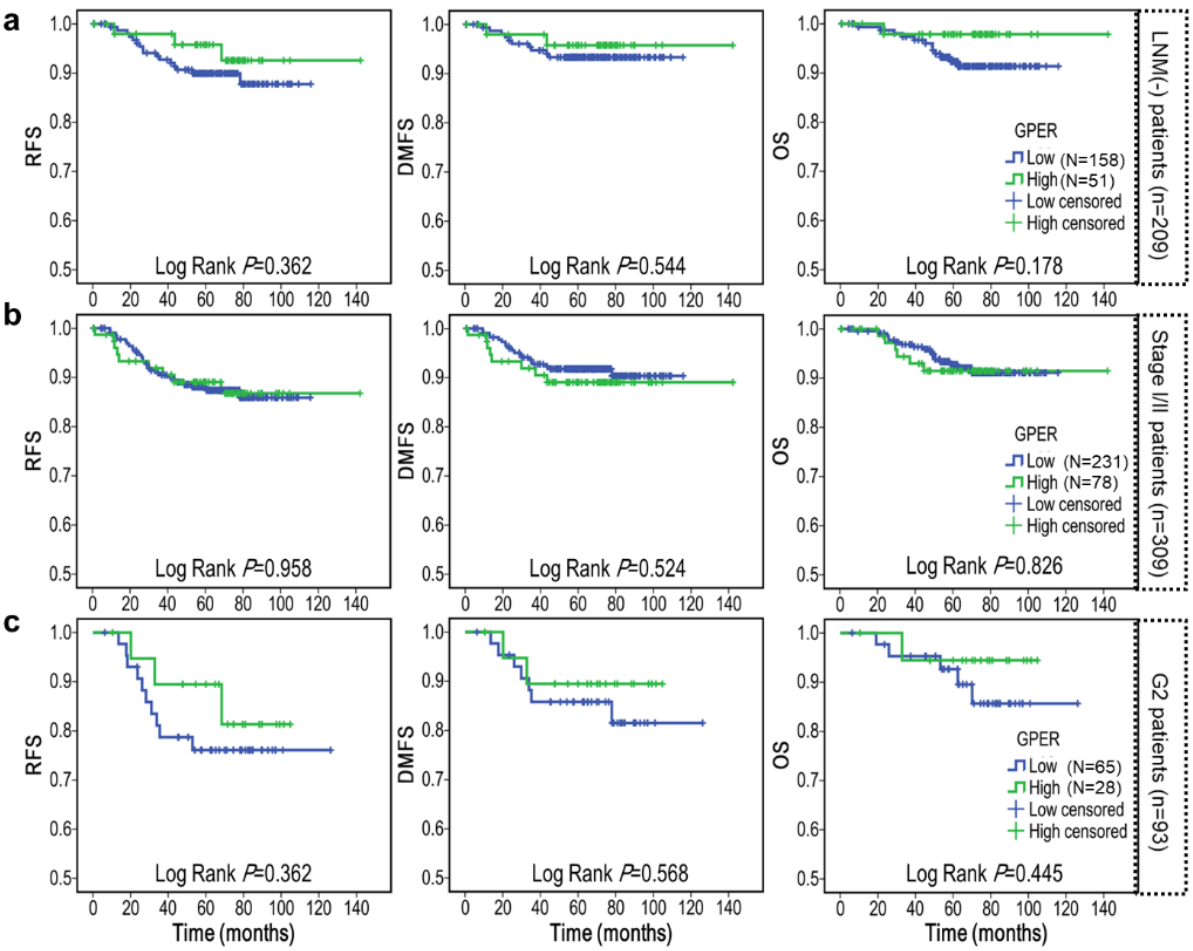

Supplementary Figure 2. Kaplan-Meier curves of RFS, DMFS, OS between GPER-low and GPER-high groups in LNM (-) patients, stage I/II patients and G2 tumor patients.
