# Supplementary material for: High GPER expression in triple-negative breast cancer is linked to pro-metastatic pathways and predicts poor patient outcomes
Source: NPJ Breast Cancer. 2022 Aug 30;8:100. doi: 10.1038/s41523-022-00472-4 (PMC9427744; doi:10.1038/s41523-022-00472-4)
Supplement: Supplementary file 2 — Reporting Summary [file 41523_2022_472_MOESM2_ESM.pdf]

nature portfolio

Corresponding author(s): Haojun Luo and Keda Yu

Last updated by author(s): Jul 5, 2022

Reporting Summary

Nature Portfolio wishes to improve the reproducibility of the work that we publish. This form provides structure for consistency and transparency in reporting. For further information on Nature Portfolio policies, see our Editorial Policies and the Editorial Policy Checklist.

Statistics

For all statistical analyses, confirm that the following items are present in the figure legend, table legend, main text, or Methods section.

n/a	Confirmed	
☐	☒	The exact sample size (*n*) for each experimental group/condition, given as a discrete number and unit of measurement
☐	☒	A statement on whether measurements were taken from distinct samples or whether the same sample was measured repeatedly
☐	☒	The statistical test(s) used AND whether they are one- or two-sided<br>*Only common tests should be described solely by name; describe more complex techniques in the Methods section.*
☐	☒	A description of all covariates tested
☐	☒	A description of any assumptions or corrections, such as tests of normality and adjustment for multiple comparisons
☐	☒	A full description of the statistical parameters including central tendency (e.g. means) or other basic estimates (e.g. regression coefficient) AND variation (e.g. standard deviation) or associated estimates of uncertainty (e.g. confidence intervals)
☐	☒	For null hypothesis testing, the test statistic (e.g. *F*, *t*, *r*) with confidence intervals, effect sizes, degrees of freedom and *P* value noted<br>*Give P values as exact values whenever suitable.*
☐	☒	For Bayesian analysis, information on the choice of priors and Markov chain Monte Carlo settings
☐	☒	For hierarchical and complex designs, identification of the appropriate level for tests and full reporting of outcomes
☐	☒	Estimates of effect sizes (e.g. Cohen's *d*, Pearson's *r*), indicating how they were calculated

Our web collection on statistics for biologists contains articles on many of the points above.

Software and code

Policy information about availability of computer code

Data collection	NA
Data analysis	All statistical analyses were done by using the SPSS standard version 25 software and Stata version 13.0 software. RNA-seq data of 360 TNBC patients was used to quantify the group difference through the R software limma package, and gene set enrichment analysis (GSEA) was applied on the ranked gene list based on group difference using clusterProfiler. Gene set variation analysis (GSVA) analysis was performed on log2 (FPKM+1) expression values by using GSVA R package. Differences between different GPER expression groups were calculated with the limma package. The "c2.cp.kegg.v7.2.symbols.gmt" gene set was downloaded from the Molecular Signatures Database.

For manuscripts utilizing custom algorithms or software that are central to the research but not yet described in published literature, software must be made available to editors and reviewers. We strongly encourage code deposition in a community repository (e.g. GitHub). See the Nature Portfolio guidelines for submitting code & software for further information.

Data

Policy information about availability of data

All manuscripts must include a data availability statement. This statement should provide the following information, where applicable:

- Accession codes, unique identifiers, or web links for publicly available datasets
- A description of any restrictions on data availability
- For clinical datasets or third party data, please ensure that the statement adheres to our policy

RNA Sequencing data that support the findings of this study have been deposited in NCBI Sequence Read Archive with the accession codes SRP157974. All other relevant data are available from the corresponding author on request.

Human research participants

Policy information about studies involving human research participants and Sex and Gender in Research.

Reporting on sex and gender	The findings apply only to females. Sex was considered in study design and only female patients' data were collected. The RNA sequencing data of 360 female patients was deposited in the NCBI Sequence Read Archive (SRA: SRP157974). Male-based analyses were not performed as male patients with triple-negative breast cancer were less than 1%.
Population characteristics	360 patients who underwent surgery at Fudan University Shanghai Cancer Center (FUSCC) between 2007 and 2014 were included in this study (Table 1). The mean age of all patients was 53.3 ± 11.4 years old (range, 25-84 years old) and 62.2% of them were post-menopause. Most tumors were pT2 (60.8%), without lymph node metastasis (LNM) (58.1%), TNM staging II (61.1%), invasive ductal carcinoma (91.7%), nuclear grade 3 (64.4%) and within the basal-like intrinsic subtype (76.9%). As for this cohort, we classified the tumors into four transcriptome-based subtypes: luminal androgen receptor (LAR) subtype (22.5%), immunomodulatory (IM) subtype (24.2%), basal-like immune-suppressed (BLIS) subtype (38.6%) and mesenchymal-like (MES) subtype (14.7%). Distant metastases were excluded present at the time of surgery. No patients received any systemic adjuvant therapy besides chemotherapy. The median follow-up interval was 67.1 months (range 0.3 to 144.2 months). At the time of analysis, 60 patients underwent recurrence, 50 patients had metastatic events and 40 patients died; the RFS was 83.3%, DMFS was 86.1% and OS was 88.9%.
Recruitment	From January 1, 2007 to December 31, 2014, primary tumor tissue and blood samples were obtained from 504 consecutive female Chinese patients with TNBC treated at Fudan University Shanghai Cancer Center (FUSCC). Among these patients, 279 had whole exome sequencing (WES) data on primary tumor tissue and paired blood samples, 401 had copy-number alteration (CNA) data and 360 had RNA sequencing data on primary tumor tissue. 360 patients with RNA sequencing data were enrolled in this study according to the following defined criteria: (1) female patients diagnosed with unilateral disease; (2) histologically confirmed the ER-α (-), PR (-) and HER2 (-) phenotype; (3) no evidence of distant metastasis at diagnosis; (4) sufficient frozen tissues available for further research.
Ethics oversight	Ethical review and approval were waived for this study, due to the data reported in this paper have been described in our published article and deposited in the NCBI Sequence Read Archive (SRA: SRP157974). All patients provided appropriate informed consent for data and tissue use.

Note that full information on the approval of the study protocol must also be provided in the manuscript.

Field-specific reporting

Please select the one below that is the best fit for your research. If you are not sure, read the appropriate sections before making your selection.

☒ Life sciences ☐ Behavioural & social sciences ☐ Ecological, evolutionary & environmental sciences

For a reference copy of the document with all sections, see nature.com/documents/nr-reporting-summary-flat.pdf

Life sciences study design

All studies must disclose on these points even when the disclosure is negative.

Sample size	360 TNBC patients with RNA sequencing data were enrolled in this study
Data exclusions	360 patients with RNA sequencing data were enrolled in this study according to the following defined criteria: (1) female patients diagnosed with unilateral disease; (2) histologically confirmed the ER-α (-), PR (-) and HER2 (-) phenotype; (3) no evidence of distant metastasis at diagnosis; (4) sufficient frozen tissues available for further research.
Replication	All in vitro experiments were performed three times.
Randomization	NA

Blinding	NA

Reporting for specific materials, systems and methods

We require information from authors about some types of materials, experimental systems and methods used in many studies. Here, indicate whether each material, system or method listed is relevant to your study. If you are not sure if a list item applies to your research, read the appropriate section before selecting a response.

Materials & experimental systems

n/a	Involved in the study	
☐	☒	Antibodies
☒	☐	Eukaryotic cell lines
☒	☐	Palaeontology and archaeology
☒	☐	Animals and other organisms
☒	☐	Clinical data
☒	☐	Dual use research of concern

Methods

n/a	Involved in the study	
☒	☐	ChIP-seq
☒	☐	Flow cytometry
☒	☐	MRI-based neuroimaging

Antibodies

Antibodies used	anti-GPER (1:250, ab39742, Abcam)
Validation	The antibodies were used according to the manufacturers' recommendation and have validated in our and others published articles (PMID: 24874276, 32030797 and 31261736).
